# Redox Balance and Inflammatory Response in Follicular Fluids of Women Recovered by SARS-CoV-2 Infection or Anti-COVID-19 Vaccinated: A Combined Metabolomics and Biochemical Study

**DOI:** 10.3390/ijms25158400

**Published:** 2024-08-01

**Authors:** Maria A. Castiglione Morelli, Assunta Iuliano, Licia Viggiani, Ilenia Matera, Alessandro Pistone, Sergio C. A. Schettini, Paola Colucci, Angela Ostuni

**Affiliations:** 1 Department of Sciences, University of Basilicata, 85100 Potenza, Italy; maria.castiglione@unibas.it (M.A.C.M.); licia.viggiani@unibas.it (L.V.); ilenia.matera@unibas.it (I.M.); alessandro.pistone@unibas.it (A.P.); 2 Center for Reproductive Medicine of “San Carlo” Hospital, 85100 Potenza, Italy; susy.iuliano@gmail.com (A.I.); sergio.schettini@ospedalesancarlo.it (S.C.A.S.); paola.colucci@ospedalesancarlo.it (P.C.)

**Keywords:** oxidative stress, inflammation, SARS-CoV-2, COVID-19, female fertility, follicular fluid, NMR-based metabolomics, biomarkers

## Abstract

To date, not many studies have presented evidence of SARS-CoV-2 infecting the female reproductive system. Furthermore, so far, no effect of the administration of anti-COVID 19 vaccines has been reported to affect the quality of oocytes retrieved from women who resorted to assisted reproduction technology (ART). The FF metabolic profiles of women who had been infected by SARS-CoV-2 before IVF treatments or after COVID-19 vaccination were examined by ^1^H NMR. Immunochemical characterization of proteins and cytokines involved in the redox and inflammatory pathways was performed. The increased expression of SOD2 and NQO1, the lack of alteration of IL-6 and CXCL10 levels, as well as the increased expression of CD39, suggested that, both sharing similar molecular mechanisms or proceeding along different routes, the redox balance is controlled in the FF of both vaccinated and recovered women compared to controls. The lower amount of metabolites known to have proinflammatory activity, i.e., TMAO and lipids, further supported the biochemical results, suggesting that the FF microenvironment is controlled so as to guarantee oocyte quality and does not compromise the outcome of ART. In terms of the number of blastocysts obtained after ICSI and the pregnancy rate, the results are also comforting.

## 1. Introduction

According to the World Health Organization (https://covid19.who.int/), the COVID-19 pandemic, produced by severe acute respiratory syndrome coronavirus 2 (SARS-CoV-2), has caused more than 775 million confirmed cases with approximately 7.0 million deaths at the time of writing (accessed on 12 May 2024). Additionally, the COVID-19 pandemic had a significant economic and health impact worldwide, affecting most medical fields, including fertility clinics. The rapidly spreading disease and the subsequent massive vaccination campaign raised some concerns regarding potential detrimental effects on human gametes and future fertility. Initially, the exclusion of pregnant women from the vaccine trials resulted in a lack of data on whether the vaccines were safe to be used before or during fertility treatment or pregnancy, and this uncertainty led to some hesitancy toward vaccination. Gradually, knowledge of SARS-CoV-2 and its transmission became available and allowed Assisted Reproduction Technology (ART) activities to resume within certain restrictions [1], until the COVID-19 vaccination finally seemed to be safe for pregnant women [2].

The potential impact of COVID-19 on reproduction, fertility care, pregnancy, and neonatal outcomes has been investigated by many authors in the last four years (i.e., [3,4,5,6,7]). Some studies reported the detrimental impact that oxidative stress has on the quality of oocytes and embryos, implying that SARS-CoV-2 infection could alter female fertility [8,9].

The effects of corona vaccination on human fertility and assisted reproduction have also been the object of study [1,10,11,12,13]. A systematic review and meta-analysis were conducted to summarize and assess the available data on the possible impact of COVID-19 vaccines on male and female fertility and, on the basis of the published studies, the authors claimed there is no scientific proof of any association between COVID-19 vaccines and fertility impairment in men or women [9].

A couple of years ago, we evaluated the effects of SARS-CoV-2 infection or vaccination against SARS-CoV-2 infection on the expression of three proteins present in follicular fluid (FF) in a small group of women who followed in vitro fertilization (IVF) treatments. These proteins were considered markers of oocyte quality, and the metabolic profile of FFs was also analyzed in healthy controls, recovered COVID-19 patients, and vaccinated women [14].

As a continuation of our previous work, in the present study, we investigated FF biomarkers that could monitor the effects of SARS-CoV-2 infection and vaccination on oocyte quality, particularly those involved in maintaining redox balance and inflammation. Furthermore, the metabolic profile of FFs was also evaluated. We first analyzed by nuclear magnetic resonance (NMR) the difference in FF metabolic profiles between women who had been infected by SARS-CoV-2 before IVF treatments and/or vaccinated, or neither. Additionally, we tested the FFs of the 55 women examined in the study for the expression of some proteins used as biomarkers of inflammation and oxidative stress; the diagnostic importance of detected biomarkers and metabolites was also evaluated; and finally, we analyzed possible relationships between NMR metabolites and clinical parameters and proteins. Considering all our data, the COVID-19 vaccine and infection from SARS-CoV-2 do not seem to have adverse effects on women’s fertility.

## 2. Results

### 2.1. Patient Data

In this study, we enrolled 55 women aged between 18 and 42 years with an FSH value < 10 IU/mL, AMH value > 1 ng/mL, antral follicle count > 8, and BMI < 30 affected by different forms of infertility before and during the coronavirus pandemic. They were selected as follows: nineteen women whose FFs were collected before the coronavirus pandemic were used as a control so that exposure both to the virus and the vaccine could be ruled out, eleven women who received vaccination against the SARS-CoV-2 infection, and twenty-five recovered COVID-19 patients. Both the vaccinated woman and those who recovered from COVID-19 performed a molecular test via an oropharyngeal swab to exclude ongoing infection at the time of oocytes retrieval. The patient’s clinical characteristics are reported in Table 1. When comparing healthy controls, vaccinated women, and recovered COVID-19 patients, we observed these differences: (i) the number of mature oocyte MII is lower in recovered COVID-19 women (3.6 ± 2.6) in comparison to healthy controls (6.9 ± 4.8) and vaccinated women (5.4 ± 3.9) (*p* = 0.019); (ii) the number of zygotes is higher in vaccinated women (4.1 ± 2.5), respect to healthy controls (1.4 ± 0.8) and recovered COVID-19 women (2.7 ± 1.7) (*p* = 0.001); (iii) although not statistically significant, the number of blastocysts is greater in the group of vaccinated patients compared to those recovered from COVID-19 and healthy controls. In fact, in the group of vaccinated patients, the blastulation rate is higher than in those recovered from COVID-19. In the group of vaccinated patients 11 embryo transfers (ET) were performed on 11 patients who underwent to oocyte retrieval, while in the group of patients who recovered from COVID-19, 17 embryo transfers were performed on 25 patients who underwent oocyte retrieval; (iv) although not statistically significant, the pregnancy rate and the live births rate per embryo transfer are higher in patients recovered from COVID-19 in comparison to vaccinated woman and healthy controls, although the abortion rate is higher.

### 2.2. NMR Results

**Figure 1:**
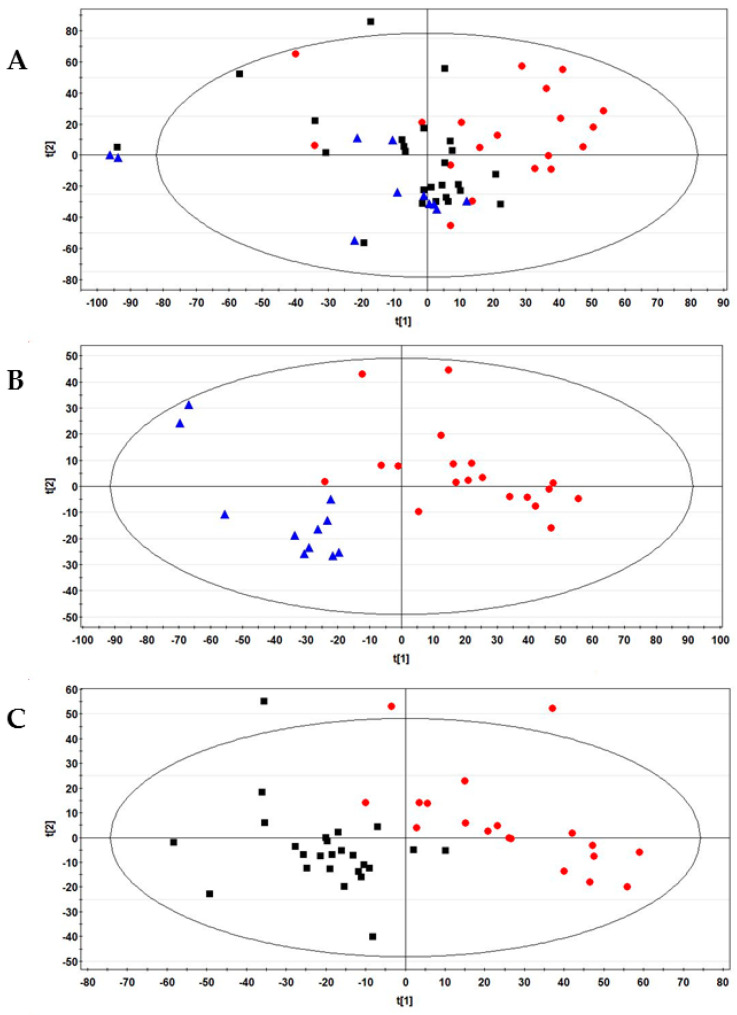
(**A**) PCA Score plot obtained from the ^1^H-NMR FF spectral data of all 55 women examined in this study. (**B**) PLS-DA score plot between control and vaccinated women. The R^2^Y and Q^2^ values for the two-component model were: 0.82 and 0.71, respectively; (**C**) PLS-DA score plot between control women and recovered COVID-19 patients. The R^2^Y and Q^2^ values for the three-component model were: 0.83 and 0.50, respectively. Data were colored by group: healthy control (N = 19, red dots), vaccinated (N = 11, blue triangles), and recovered COVID-19 (N = 25, black boxes).

To identify the differential metabolites among the examined groups of women, an exploratory analysis of the ^1^H NMR data collected from their FFs was first created using principal component analysis (PCA). A three-component model was obtained with cumulative R^2^ and Q^2^ values of 0.59 and 0.35, respectively. Figure 1A reports the PCA score plot where the healthy controls (plotted in red) clustered on the right side of the diagram and the SARS-CoV-2 vaccinated (in blue) and recovered COVID-19 women (black) on the left side.

Partial least squares discriminant analysis (PLS-DA) confirmed the differences observed by unsupervised analysis. The model reported in Supplementary Figure S1 shows that healthy women and the groups formed by vaccinated and previously positive women were positively discriminated against.

In addition, we investigated the effects on FFs of vaccination against SARS-CoV-2 or COVID-19 infection by pair-wise comparisons of these classes with respect to healthy controls using PLS-DA. The results are reported in Figure 1B,C, respectively; the two models were validated by a permutation test (Figure S2). PLS-DA also generated a list of 24 signals with VIP (variable importance in the projection) values > 1, corresponding to 16 potential metabolites for discriminating the three examined groups (Table S1).

**Figure 2:**
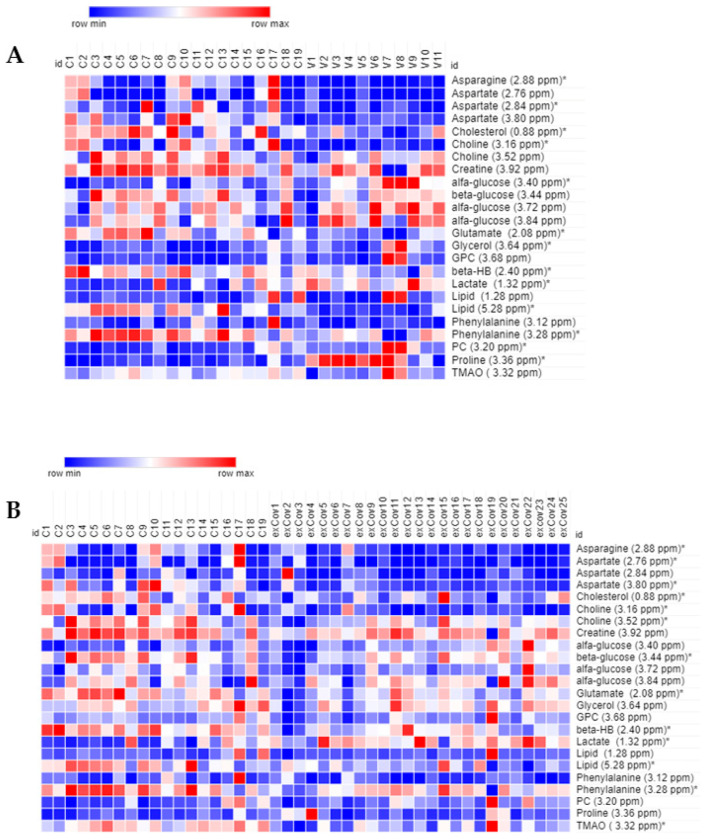
Heat map of the most relevant metabolites (with a VIP value > 1) associated with the differentiation between control (N = 19, labeled C) and: (**A**) vaccinated (N = 11, V) and (**B**) recovered COVID-19 women (N = 25, exCov). Rows: quantification of NMR integral bin regions of metabolites with VIP > 1. Columns: different groups of women. The color scale indicates values ranging from blue (the lowest) to red (the highest). * *p* < 0.05.

A panel of 13 metabolites resulted significantly differently in the group of vaccinated women in comparison to healthy controls: the levels of glucose, glycerol, lactate, phoshocholine, and Pro were higher while those of the amino acids Asn, Asp, Glu, and Phe were lower, together with cholesterol, choline, β-hydroxybutyrate, and lipids (Figure 2A).

The group of recovered COVID-19 patients was characterized by a higher level of lactate and significantly lower levels of Asn, Asp, Glu, Phe, cholesterol, choline, lipids, β-hydroxybutyrate, and TMAO (Figure 2B).

### 2.3. Evaluation of Oxidative Stress and Antioxidant Capacity

**Figure 3:**
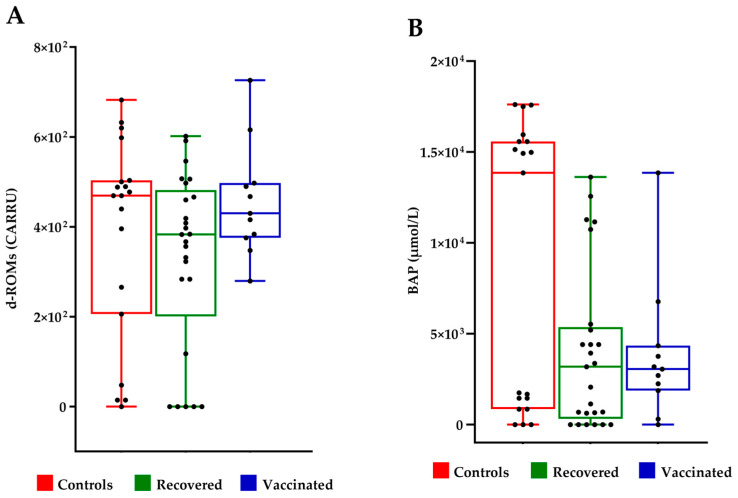
Evaluation of oxidative stress in follicular fluids. (**A**) Measure of hydroperoxide level by d-ROMS assay; UCARR indicate “Carratelli Unit”, an arbitrary unit, and 1 U CARR corresponds to the color development caused by a H_2_O_2_ solution at a concentration of 0.08%; (**B**) measure of the antioxidant power by BAP test. The results were analyzed with Wilcoxon–Mann–Whitney test.

The evaluation of oxidative stress and antioxidant capacity of FFs was conducted using the d-ROMs test and the BAP test, respectively. The d-ROMs test measures the hydroperoxide content. The BAP test measures the antioxidant level. The d-ROMs and BAP tests, when used together, offer a thorough assessment of oxidative stress. No significant differences were observed in terms of oxidative stress between the FF of controls, recovered COVID-19 and vaccinated women (Figure 3).

### 2.4. Expression Analysis of Oxidative Stress Biomarkers

Oxidative stress and inflammation can negatively affect the maturation and quality of oocytes. In order to understand the molecular mechanisms underlying the apparent redox balance both in the FF of vaccinated women and in patients recovered from COVID-19, we conducted a series of investigations on the expression of some proteins implicated in maintaining the redox balance and, therefore, indirectly in controlling inflammatory processes.

Nuclear factor erythroid 2-related Factor 2 (NRF2) is a transcription factor that regulates cellular defense against toxic and oxidative insults through the expression of genes coding for antioxidant enzymes and cytoprotective proteins, including NADPH quinone oxidoreductase 1 (NQO1) and superoxide dismutase (SOD2), both involved in the response to oxidative stress and, indirectly, in limiting inflammatory processes [15,16].

**Figure 4:**
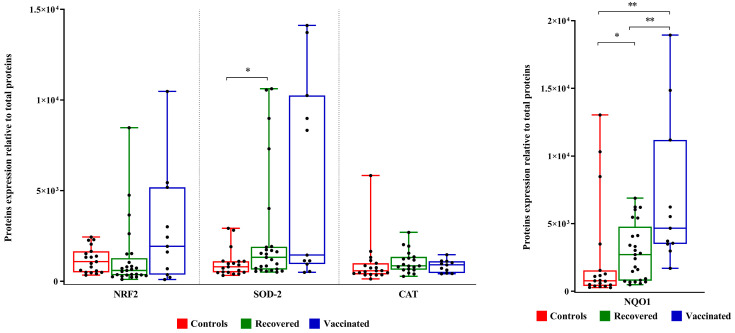
NRF2, SOD2, catalase, and NQO1 expression levels in FFs. Densitometric analysis of immunoreactive bands measured by Western Blot; protein levels were normalized to total protein, and data are expressed, as mean ± standard deviation (SD) and analyzed with Wilcoxon–Mann–Whitney test (* *p* < 0.05, ** *p* < 0.01).

Although there is no statistical difference in the expression levels of NRF2, it is interesting to note an increase in the expression of SOD2, which is statistically significant in recovered women. Although not statistically significant, the expression of catalase, on average, increased in the FFs of vaccinated and recovered women. Increased expression of NQO1 in the groups of vaccinated and recovered women compared to controls was observed (Figure 4).

### 2.5. Analysis of Biomarkers of Inflammation

To analyze the specific effect of COVID-19 infection and vaccine on the inflammatory potential of FFs, some proinflammatory cytokine and chemokine levels were quantified using ELISA sandwich assays.

**Figure 5:**
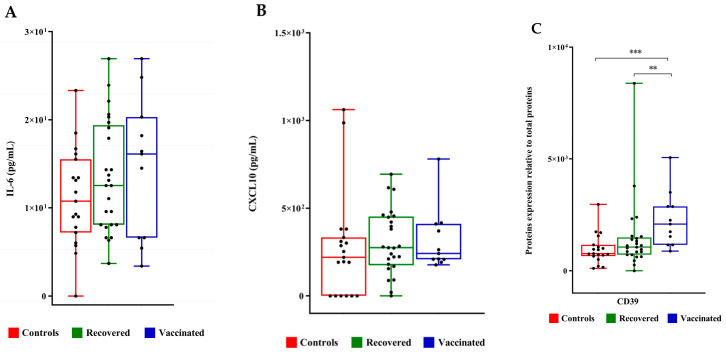
IL6 and CXCL10 concentrations and CD39 expression levels in follicular fluids. (**A**) IL-6 and (**B**) CXCL10 concentrations were measured by ELISA sandwich. (**C**) CD39 expression level was evaluated by densitometric analysis of the immunoreactive band obtained by Western Blot; protein levels were normalized to total protein, and data are expressed as mean ± standard deviation (SD) and analyzed with Wilcoxon–Mann–Whitney test (** *p* < 0.01, *** *p* < 0.001).

No significant differences were observed between IL-6 as well as CXCL10 concentrations in the three FF groups (Figure 5A,B). TNF-α was not detected at all in the analyzed FFs. Furthermore, we evaluated the expression level of CD39 (ectonucleoside triphosphate diphosphohydrolase-1, ENTPD1) involved in several pathophysiological events, among them infections, the control of the immune response, vascular inflammation, and thrombosis [17]. The expression level of CD39 increased in the FFs of vaccinated and recovered COVID-19 patients compared to that of controls (Figure 5C).

### 2.6. Evaluation of the Diagnostic Importance of Detected Biomarkers and Metabolites

**Figure 6:**
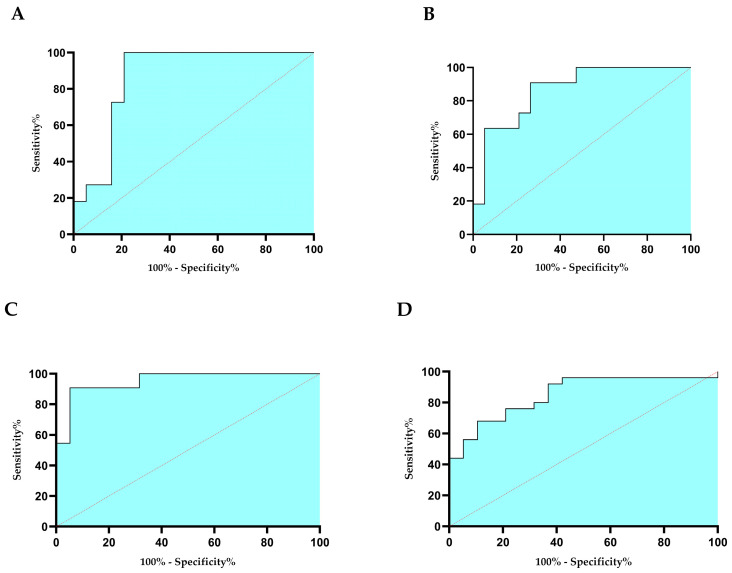
ROC curves for NQO1 (**A**), CD39 (**B**), lipids (**C**), and TMAO (**D**). Sensitivity indicates the true positive, and 100% specificity indicates false positives. The curves were obtained with GraphPad Prism 8.0 software.

For the evaluation of the diagnostic importance of those biomarkers that were significantly different between controls and patients, ROC curve analysis was performed (Figure 6). AUC of 0.87 and *p* values of 0.001 were found for both NQO1 (Figure 6A) and CD39 (Figure 6B), thus suggesting that they are good markers for discriminating the FFs of controls compared to those of vaccinated women.

From the ROC analysis of the metabolites, which resulted in significantly different in the FF of controls compared to patients, lipids in vaccinated women (Figure 6C) (AUC 0.95, *p* < 0.0001) and TMAO (Figure 6D) in women recovered from SARS-CoV-2 infection (AUC 0.85, *p* < 0.0001) were found.

### 2.7. Relationships between NMR Variables with Clinical Parameters and Proteins

Metabolites identified by NMR analysis revealed some significant correlations, both with clinical parameters and proteins.

In recovered COVID-19 women, choline was positively correlated with AMH (r = 0.48, *p* = 0.016), FSH (r = 0.51, *p* = 0.009), and progesterone (r = 0.51, *p* = 0.01). TMAO was negatively correlated with the number of MII oocytes (r = 0.42, *p* = 0.038). Glucose was positively correlated with FSH (r = 0.53, *p* = 0.006) and progesterone (r = 0.49, *p* = 0.016). Glutamate was positively correlated with progesterone (r = 0.44, *p* = 0.029). Furthermore, phenylalanine was negatively correlated with SOD2 (r = −0.412, *p* = 0.041).

In vaccinated women, a positive correlation was found between glycerol and the number of zygotes (r = 0.62, *p* = 0.04), MII oocytes (r = 0.73, *p* = 0.013), and AMH (r = 0.65, *p* = 0.033). Choline correlates with MII oocytes (r = 0.71, *p* = 0.016). Lipids correlate with progesterone (r = 0.72, *p* = 0.016) and AMH (r = 0.72, *p* = 0.016). AFC correlates with aspartate (r = 0.76, *p* = 0.008), and TMAO (r = 0.66, *p* = 0.031). Finally, aspartate was negatively correlated with CD39 (r = −0.75, *p* = 0.009).

## 3. Discussion

Angiotensin-converting enzyme 2 (ACE2), a pivotal component within the renin-angiotensin system, serves as the primary cellular receptor for the spike protein of SARS-CoV-2. ACE2 can be found at the surface of many cell types, including the ovary, uterus, vagina, and placenta [18]. It regulates steroidogenesis, folliculogenesis, oocyte maturation, and ovulation (for references, see [19]); therefore, SARS-CoV-2 infection represents a potential risk to reproductive health [20,21]. A small number of studies evaluated the impact of both SARS-CoV-2 infections, sometimes with conflicting results; even fewer studies have evaluated the impact of COVID-19 vaccination on oocytes and embryos during IVF cycles [22,23,24,25,26].

Human follicular fluid (FF) is a specialized fluid found within ovarian follicles containing plasma exudates, granulosa cell metabolic products, plasma proteins, various hormones, and paracrine growth factors. It plays a crucial role in supporting oocyte growth, maturation, and development, allowing an interplay between theca and granulosa cells with oocyte [27]. It follows that any alteration of the microenvironment, whether at a local or systemic level, can have repercussions on the development and quality of the oocyte. Obtaining follicular fluid is a simple and noninvasive method, making it ideal for assessing the developmental potential of oocytes by evaluating oxidative stress levels within the follicular environment [28].

Oxidative stress is the term used to indicate the imbalance between reactive oxygen species (ROS) and antioxidant defense mechanisms. Inflammatory processes are closely related to oxidative stress and vice versa, and it is not easy to understand which is established first. ROS are naturally generated during ovulation [29,30]. Oxidative stress and inflammation can negatively affect oocyte maturation and quality and consequently could have direct effects on the efficiency of medically assisted procreation techniques [31]. It is well known that during infection of host cells, coronaviruses alter the redox balance and consequently induce inflammation in patients with COVID-19 disease [32,33]. To the best of our knowledge, few reports have addressed the molecular mechanism that could be involved in the potential dysregulation of the redox balance and/or the metabolomic profiling of the follicular microenvironment [14,34].

In the present study, we investigated the effects of SARS-CoV-2 infection and anti-COVID-19 vaccination on the follicular fluids of women who underwent medically assisted procreation techniques between 2019 and 2023. Supposedly, no significant differences were observed in terms of oxidative stress between the FF of controls, recovered COVID-19 and vaccinated patients. On the other hand, none of the women had shown significant side effects after administration of the vaccine, nor had those who had contracted the infection shown serious clinical symptoms. However, we decided to further investigate whether any of the mechanisms that supervise or intervene in redox and inflammatory processes may have been affected.

Metabolomics investigates all low-molecular-weight molecules produced in cells, tissues, and biofluids [35]. In particular, the study of follicular fluid by metabolomics approaches makes it possible to understand the nutritional environment and requirements of the oocyte, and it may also lead to the identification of potential biomarkers of oocyte quality.

Combining metabolomics with biochemical studies leads to a deeper understanding of disease mechanisms, enriches biomarker research by validating and characterizing them, and also enhances diagnostic and prognostic approaches.

The transcription factor NRF2 regulates the expression of several genes involved in cryoprotection towards oxidative stress and inflammation [15] including superoxide dismutase 2 (SOD2), a mitochondrial protein that converts superoxide anions to hydrogen peroxide and oxygen, and NQO1, an antioxidant enzyme that uses NADH or NADPH as substrates to reduce quinones to hydroquinones while avoiding the formation of highly reactive semiquinones [16]. Although we do not observe any significant changes in NRF2 expression levels, the increased expression of SOD2 and NQO1 suggested that oxidative stress is controlled in FFs of both vaccinated and recovered women. Enhanced proinflammatory cytokine and chemokine levels are the hallmarks of SARS-CoV-2 infection [36,37]. In FFs of the three groups of examined women, we did not detect any changes in the levels of proinflammatory cytokine IL6, considered among the main culprits in the pathogenesis of the inflammatory cascade following SARS-CoV-2 infection, nor of the cytokine CXCL10, associated with the cytokine storm induced by SARS-CoV-2 infection as well as locally produced by granulosa cells [38].

ROC curves analysis showed an excellent diagnostic capacity of both CD39, NQO1 and lipids as biomarkers that allow to distinguish FFs of vaccinated women from those of controls. Moreover, TMAO could be considered an excellent biomarker to identify FF of women recovered from SARS-CoV-2 infection.

Inflammation and thrombosis are closely intertwined processes, involving key players such as platelets, innate immune cells, and endothelial cells. CD39, TMAO, and lipid biomarkers are intricately linked in the contexts of inflammation, oxidative stress, and thrombosis. CD39 exhibits anti-inflammatory and antithrombotic roles, helping to stabilize lipid profiles and inhibit platelet aggregation [17]. Conflicting studies have been reported on the role of plasma soluble CD39 as a marker of vascular damage in patients with COVID-19 [39]. Interestingly, we found a greater expression level of soluble CD39 in both FFs of vaccinated and recovered COVID-19 women, compared to controls, supporting the activation of molecular mechanisms that control oxidative stress in patient FFs.

TMAO, a metabolite derived from the dietary intake of choline, phosphatidylcholine, and carnitine, metabolized by gut microbiota promotes inflammation, oxidative stress, and thrombosis by enhancing platelet reactivity and inducing inflammatory pathways [40]. It has been suggested that gut TMAO has the potential to increase COVID-19 disease severity [41]. NMR analysis indicates a decrease in TMAO in the FFs of women recovered from infection. Lipid biomarkers reflect the state of lipid metabolism and are influenced by both inflammatory processes and thrombotic risk factors also detected in patients who have contracted SARS-CoV-2 infection [42]. NMR analysis of FFs indicates a decrease in lipids in the FFs of both recovered COVID-19 and vaccinated women.

From the correlation analysis between NMR variables with proteins in FFs of vaccinated women emerges that CD39 is negatively correlated to aspartate. Although there is no direct interaction between CD39 and aspartate, their functions are interconnected through nucleotide metabolism and purinergic signaling. Aspartate is essential for the synthesis of nucleotides [43], which are substrates for CD39, linking it to the role of CD39 in modulating extracellular nucleotide levels and related inflammatory and immune response pathways. Therefore, the results further support that vaccination does not appear to have caused alterations in the inflammatory state of FFs.

The analysis of the clinical ICSI outcome shows that vaccinated women have a reduction in the number of MII oocytes (5.4 ± 3.9) compared to women recovered from COVID-19 (3.6 ± 2.6) and healthy controls (6.9 ± 4.8), and that they have a miscarriage rate similar to the controls (20%) but higher than patients recovered from COVID-19 (11.1%). These data show that patients that recovered from COVID-19 have a lower blastulation rate than vaccinated patients and controls, but that they have a better pregnancy rate (56% vs. 45.5% and 31.3%) and live births (47% vs. 36.4% and 25%) with fewer abortion rates (11% vs. 20%) than the other two groups. These data confirm that COVID-19 disease does not change the outcomes of the assisted reproduction technique in terms of pregnancy rates and live births.

In conclusion, our results obtained by a combination of metabolomics and biochemical analyses suggested possible markers in the FFs of patients who contracted COVID-19 and of women who were vaccinated before undertaking the medically assisted procreation process. These data also allow us to suggest and reassure that both the COVID-19 vaccine and infection with SARS-CoV-2 did not have adverse effects in terms of inflammation and/or thrombosis that could have influenced the quality of oocytes.

## 4. Materials and Methods

### 4.1. Study Participants

Fifty-five women undergoing treatment for IVF at Centre for Reproductive Medicine of “San Carlo” Hospital were selected for this study. A first group consisted of nineteen healthy women who were selected prior to the start of coronavirus pandemic, from January to December 2019, and they were used as a control group. These women presented mild or moderate male infertility factors (8) or unexplained infertility (11). A second group consisted of 11 women who were vaccinated against SARS-CoV-2 infection. The median time from vaccine dose to recruitment and sampling was 45 days (range 18–365 days). A third group consisted of 25 women who were SARS-CoV-2 affected but became fully negative at the time of IVF, as confirmed by a molecular test; their mild clinical symptoms are reported in Table S2. The median time between the infection of the patients with SARS-CoV-2 and the retrieval of FF was 4 months (interval: 1–13 months). The women from the second and third groups were selected from March 2022 to April 2023. The infertility indications for the group of vaccinated women were: 6 male infertility factors, 1 unexplained infertility, and 4 tubaric diseases. For the group of recovered COVID-19 patients, the infertility indications were: 6 male infertility factors, 13 unexplained infertility, 5 tubaric disease, and 1 endometriosis. Clinical characteristics of all 55 patients are described in Table 1.

Written informed consent was obtained from all participants enrolled in the study that was approved by the local ethical committee (Comitato Etico Unico Regionale per la Basilicata, approval number: 20210053801 of 16 February 2022).

All patients performed ovarian reserve tests: basal FSH, AMH, and antral follicle count before starting ovarian stimulation to have homogeneous samples with respect to the ovarian reserve.

Participants received stimulation with recombinant follicle-stimulating hormone (FSH) (Gonal-f, Merck Serono (Roma, Italy) or Ovaleap, Theramex (Milano, Italy) or urinary highly purified FSH (Fostimon, IBSA, Lodi, Italy) and gonadotropin-releasing hormone (GnRH) antagonists (Cetrotide, Merk Serono (Roma, Italy) or Fyremadel, Ferring (Milano, Italy). In particular, the follicular stimulation was started on day 2 of the menstrual cycle with a FSH dose calculated according to the nomogram of La Marca. The follicular growth was monitored with ultrasound scans and estradiol and progesterone assessments, first on day 5 and then every 2 days. Daily administration of a GnRH antagonist was started when the leading follicle was 14 mm in diameter and continued until the day of the trigger of the ovulation. When at least two follicles had reached 17–18 mm in diameter, ovulation was triggered with a single subcutaneous bolus of 10.000 UI of highly purified hCG (Gonasi HP 10.000, IBSA, Lodi, Italy) or 0.2 mL of triptorelina (Decapeptyl 0.1, IPSEN). The oocyte retrieval was performed after 34–36 h. The collection of cumulus-oocyte complexes and follicular fluid was performed via transvaginal.

In order to prevent the fertilization technique from affecting the ART outcomes, all the oocytes were fertilized with ICSI. Embryos were transferred into the uterus only on the fifth day, under ultrasound guidance, using soft or rigid catheters. Blastocysts were deposited at 1.5 cm from the uterus fundus. The experimental design of the study is reported in Figure S3.

### 4.2. NMR Sample Analysis

The aspirated FF was centrifuged at 10,000 rpm for 10 min to remove erythrocytes and leukocytes. The supernatant was collected and maintained frozen at −80 °C until processing. Only FF samples not contaminated by the flushing medium during the aspiration procedure were used in the analysis.

Samples for NMR analyses were prepared as described elsewhere [34,44]. All ^1^H NMR spectra were acquired at 25 °C on a Varian Inova 500 MHz spectrometer (Varian, Palo Alto, CA, USA); a Carr-Purcell-Meiboom-Gill (CPMG) pulse sequence was used. Further details were reported before [14,34,44].

All the NMR spectra were processed with the software ACD/1D NMR Processor (version 12.01, Academic Edition, ACD Labs, Toronto, ON, Canada), and integral buckets of 0.04 ppm were produced. The TSP signal and the region of 4.7–5.1 ppm around the water signal were excluded. The area of all bins was then normalized to the total spectrum area. For metabolomics assignments, we used data from literature or publicly available metabolite databases, such as the human metabolite database (HMDB, http://www.hmdb.ca/), and the biological magnetic resonance data bank (BMRB, http://www.bmrb.wisc.edu/metabolomics/) (accessed on 15 March 2024).

### 4.3. Multivariate Analysis

NMR data were imported into the program SIMPCA-P+ (version 12, Umetrics, Umeå, Sweden) and subjected to pretreatment with Pareto scaling (/√SD), which automatically mean-centers the data. First, an unsupervised Principal Component Analysis (PCA) model was built on the entire data set.

Supervised models were then built with latent structure-discrimination analysis (PLS-DA) using different groups of women: a first group of nineteen healthy participants who were examined before the COVID-19 pandemic; a second group consisted of eleven participants with vaccination against SARS-CoV-2 infection; a third group consisted of twenty-five recovered COVID-19 patients. The overall quality of the models obtained from PLS-DA was evaluated by the R^2^ and Q^2^ values, where R^2^ measures the goodness of fit and displays the explained variation by components, and Q^2^ provides an indication of the goodness of the predicted model. The PLS-DA models were validated using permutation tests.

The heatmaps reported in Figure 2 were calculated with Morpheus software, https://software.broadinstitute.org/morpheus (accessed on 1 April 2024).

### 4.4. Oxidative Balance Evaluation

Pro-oxidant status and antioxidant status evaluations were performed with d-ROMs (derivative reactive oxygen metabolites) and BAP (Biological Antioxidant Potential) tests, respectively, according to the manufacturer’s instructions (Diacron International Srl Grosseto, Italy).

### 4.5. Western Blot Analysis

FFs were diluted 1:5 in RIPA Lysis Buffer (Merk, Darmstadt, Germany) and Protease Inhibitor Cocktail (Sigma, Saint Louis, MO, USA). A total of 5 μg protein from each sample was separated by 15% SDS-PAGE and electrotransfered to Amersham^TM^ Protran^TM^ Nitrocellulose Blotting Membrane (GE Healthcare, Merk, Darmstadt, Germany). Membranes were stained with Ponceau S solutions (Sigma, Saint Louis, MO, USA) for 15 min then rinsed with distilled water and blocked with 5% milk for 1 h at room temperature. Finally, membranes were incubated overnight at 4 °C using the following primary antibodies: 1:1000 anti-NRF2 (16396-1-AP, Proteintech Europe, Manchester, UK), 1:1000 anti-SOD2 (66474-1-Ig, Proteintech Europe, Manchester, UK), 1:100 anti-NQO1 (sc-32793 Santa Cruz Biotechnology, Inc., Heidelberg, Germany), 1:1000 anti-CD39 (19229-1-AP, Proteintech Europe, Manchester, UK), and then with 1:5000 Goat Anti-Rabbit IgG Peroxidase Conjugate (A9169, Sigma, Saint Louis, MO, USA) or 1:2500 Goat anti-Mouse IgG (H + L), Superclonal™ Recombinant Secondary Antibody (A9917 Thermo Fisher Scientific, Inc., Waltham, MA, USA), for 1 h at room temperature. Signals were visualized by ECL™ Western Blotting Detection Reagents (Amersham Bioscience, Buckinghamshire, UK) or ECL WEST FEMTO PLUS—ECL-2002 (Immunological Sciences, Rome, Italy). Images were captured with Chemidoc™ XRS detection system (BioRad, Hercules, CA, USA) equipped with ImageLab 5.1 software for image acquisition and processed using GelAnalizer 2010 software (Istvan Lazar, www.gelanalyzer.com, accessed on 15 January 2024). Band intensities were normalized with respect to the total proteins evaluated after membrane staining with Ponceau Red.

### 4.6. ELISA

A solid phase sandwich enzyme linked-immunosorbent assay (Sandwich Elisa) was used to measure CX-CL10 (KE00128), IL-6 (KE00139). FFs were diluted 1:2 in sample diluent and then processed according to the manufacturer’s instructions (Proteintech, 6 Atherton Street, M3 3GS, Manchester, UK).

### 4.7. Statistical Analysis

Clinical data were compared among the three groups by univariate ANOVA (Systat 11.0, Systat Software, Inc., San Jose, CA, USA). A post-hoc Bonferroni test was performed to evaluate the significance of the observed differences. The minimum level of statistical significance was *p* < 0.05. Values are presented as mean ± standard deviation (SD).

Data from Western Blot and ELISA analyses were analyzed using GraphPad Prism 8.0 software and presented as mean ± standard deviation of three independent experiments. The normality of the data distribution was tested with Shapiro–Wilk test. Wilcoxon–Mann–Whitney test was performed to obtain pairwise comparison groups. The minimum level of statistical significance was *p* < 0.05.

### 4.8. ROC Analysis

In order to identify those markers that best discriminate the FF profile of controls compared to patients, a receiver operating characteristics (ROC) analysis was carried out on the proteins that were found statistically significant to be underexpressed or overexpressed in recovered or vaccinated patients in comparison to controls. Data were analyzed by GraphPad Prism 8.0 software with Wilson/Brown correction.

### 4.9. Correlation Tests

The correlation between NMR metabolites and biochemical markers was analyzed by GraphPad Prism 8.0 software using the Spearman’s correlation test. Significance was assumed whenever *p* < 0.05.

## Figures and Tables

**Table 1:** Clinical data of the 55 women participating in the study.

	Control ^§^	SARS-CoV-2 Vaccinated	Recovered COVID-19
Number of patients	19	11	25
Age (years)	36.9 (3.8)	35.7 (4.4)	37.1 (4.3)
FSH (UI/mL)	7.1 (1.7)	6.7 (2.6)	6.5 (2.6)
AMH (ng/mL)	5.3 (3.6)	3.2 (2.9)	2.4 (1.2)
AFC	14.2 (3.2)	15.1 (7.5)	11.4 (8.3)
Estradiol (pg/mL)	1675.0 (1065.5)	1972.9 (1250.1)	1763.0 (132.9)
Progesterone (ng/mL)	1.3 (0.7)	1.3 (0.9)	1.3 (0.3)
BMI (kg/m^2^)	22.5 (3.4)	23.9 (4.1)	23.8 (5.3)
Follicles monitored	9.8 (4.9)	11.3 (5.9)	9.5 (6.9)
Total oocytes collected	8.2 (5.0)	7.5 (3.9)	5.6 (4.3)
MII oocytes *	6.9 (4.8)	5.4 (3.9)	3.6 (2.6)
Zygotes *	1.4 (0.8)	4.1 (2.5)	2.7 (1.7)
Blastocysts	1.3 (0.7)	2.3 (2.4)	1.4 (1.2)
Pregnancy rate %	31.3 (5/16)	45.5 (5/11)	56 (9/17)
Pregnancy loss %	20 (1/5)	20 (1/5)	11.1 (1/9)
Live Birth Rate per ET %	25 (4/16)	36.4 (4/11)	47 (8/17)

Data are presented as mean values, and the standard deviation is reported in parentheses. ^§^ healthy pre-COVID; FSH = follicle stimulating hormone; AMH = antimullerian hormone; AFC = antral follicle count; BMI = body mass index; zygote = single, diploid cell formed immediately after the fertilization of an oocyte by a sperm; blastocysts = stage of embryonic development at 5 days after fertilization. * The number of MII oocytes was significantly different in different groups (*p* = 0.019) as well as the number of zygotes (*p* = 0.001); pregnancy rate = βhCG-positive/embryo transfer number; pregnancy loss rate = abortion number/pregnancy number; live birth rate per ET % = number of children born/number of transfers.

## Data Availability

The data presented in this study are available upon request from the corresponding author.

## Supplementary Materials

The following supporting information can be downloaded at: https://www.mdpi.com/article/10.3390/ijms25158400/s1.
