# Supplementary material for: Redox Balance and Inflammatory Response in Follicular Fluids of Women Recovered by SARS-CoV-2 Infection or Anti-COVID-19 Vaccinated: A Combined Metabolomics and Biochemical Study
Source: Int J Mol Sci. 2024 Aug 1;25(15):8400. doi: 10.3390/ijms25158400 (PMC11313332; doi:10.3390/ijms25158400)
Supplement: Supplementary file 1 [file ijms-25-08400-s001.zip › Table S1_Table S2.pdf]

Table S1. Some metabolites identified by NMR in follicular fluids of healthy control, women vaccinated against SARS-CoV-2 and recovered COVID19 patients.

Metabolite	NMR (δ)	Healthy (n=19)	Vaccinated (n=11)	VIP	Recovered (n=25)	VIP	p-value
Asn	2.88	277.7 ± 243.5	50.0 ± 58.9	3.4	115.5 ± 126.6	3.4	0.001
Asp	2.76	69.3 ± 70.4	30.6 ± 19.0	1.2	33.3 ± 28.6	1.5	0.02
Asp	2.84	78.1 ± 65.8	30.5 ± 24.0	1.5	52.4 ± 73.2	-	ns
Asp	3.80	349.9 ± 128.5	222.3 ± 28.3	2.7	232.0 ± 52.1	3.3	0.0001
Cholesterol	0.88	125.4 ± 35.7	86.7 ± 29.7	1.4	99.8 ± 38.4	1.2	0.01
Choline	3.16	282.4 ± 215.9	78.2 ± 54.1	3.3	118.8 ± 111.2	3.7	0.0001
Choline	3.52	220.3 ± 51.8	196.3 ± 44.0	-	190.9 ± 41.2	1.4	ns
Creatine	3.92	261.6 ± 57.9	220.0 ± 94.2	1.3	235.1 ± 50.7	1.2	ns
α–Glucose	3.40	130.5 ± 42.8	212.2 ± 71.5	2.3	159.9 ± 55.7	1.2	0.001
α –Glucose	3.44	303.7 ± 73.3	291.2 ± 58.1	-	260.4 ± 62.8	1.6	ns
α–Glucose	3.72	291.7 ± 51.9	323.0 ± 65.8	1.1	277.8 ± 59.1	1.0	ns
α–Glucose	3.84	224.4 ± 52.9	260.7 ±63.8	1.1	242.0 ± 49.5	-	ns
Glu	2.08	135.2 ± 30.1	103.0 ± 18.3	1.3	103.7 ± 26.6	1.7	0.0002
Glycerol	3.64	64.1 ± 12.8	79.6 ± 27.1	1.0	61.3 ± 15.2	-	0.02
GPC	3.68	115.2 ± 29.1	152.5 ±88.3	1.5	104.1 ± 39.9	-	0.04
β–HB	2.40	95.2 ± 17.9	77.1 ± 15.1	1.0	71.3 ± 19.0	1.5	0.0002

Lactate	1.32	353.5 ± 204.6	586.3 ±160.4	3.6	619.9 ± 181.0	5.0	0.0001
Lipid	1.28	140.2 ± 99.7	152.6 ±147.3	2.0	113.8 ± 84.0	1.4	ns
Lipid	5.28	77.1 ± 26.4	32.7 ± 13.4	1.8	54.5 ± 22.1	1.3	0.0001
Phe	3.12	92.8 ± 80.4	53.8 ± 31.7	1.2	62.5 ± 46.6	1.0	ns
Phe	3.28	244.4 ± 70.7	157.3 ± 73.7	2.1	192.1 ± 62.5	1.9	0.003
PC	3.20	110.1 ± 57.9	187.6 ± 107.1	2.2	123.1 ± 57.5	-	0.014
Pro	3.36	67.0 ± 19.4	161.4 ± 56.8	3.0	82.6 ± 40.8	-	0.0001
TMAO	3.32	45.6 ± 5.2	41.1 ± 12.8	-	36.6 ± 8.3	1.0	0.005

The average integrals of the NMR bin regions ± standard deviations are reported.

δ, NMR chemical shift

VIP, **V**ariable **I**mportance in the **P**rojection

-, VIP value < 1

p values were obtained by ANOVA. *ns* = p-value > 0.05

GPC, Glycerophosphocholine

β–HB, β–hydroxybutyrate

PC, Phosphocholine

TMAO, trimethylamine oxide

Table S2 - Symptoms presented by SARS-CoV-2 positive patients before undergoing IVF treatments.

**Symptoms**	**SARS-CoV-2 patients (N =25)**
Fever	12
Pharyngitis	2
Arthralgia	7
Aphonia	2
Cough	4
Cold	6
Headache	2
Myalgia	2
Weakness	3
Odour-blindness	6
