# Supplementary figures and images for: Redox Balance and Inflammatory Response in Follicular Fluids of Women Recovered by SARS-CoV-2 Infection or Anti-COVID-19 Vaccinated: A Combined Metabolomics and Biochemical Study
Source: Int J Mol Sci. 2024 Aug 1;25(15):8400. doi: 10.3390/ijms25158400 (PMC11313332; doi:10.3390/ijms25158400)

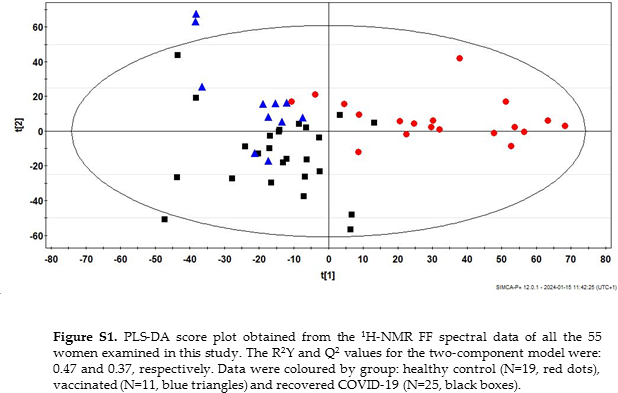

Supplement: Supplementary file 1 [file ijms-25-08400-s001.zip › Figure S1.tif]

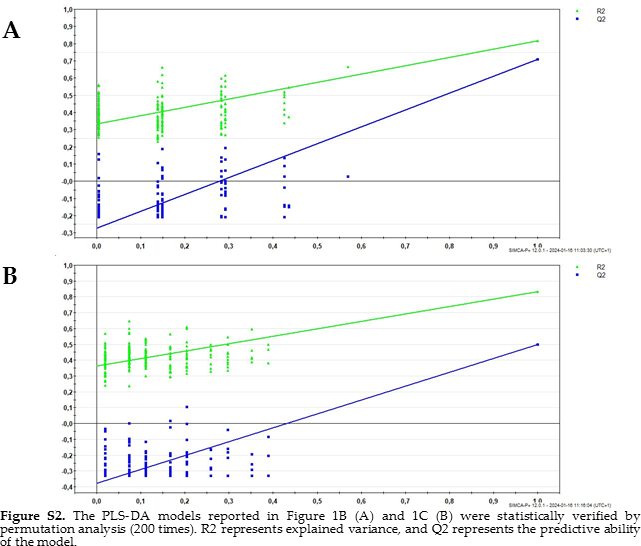

Supplement: Supplementary file 1 [file ijms-25-08400-s001.zip › Figure S2.tif]

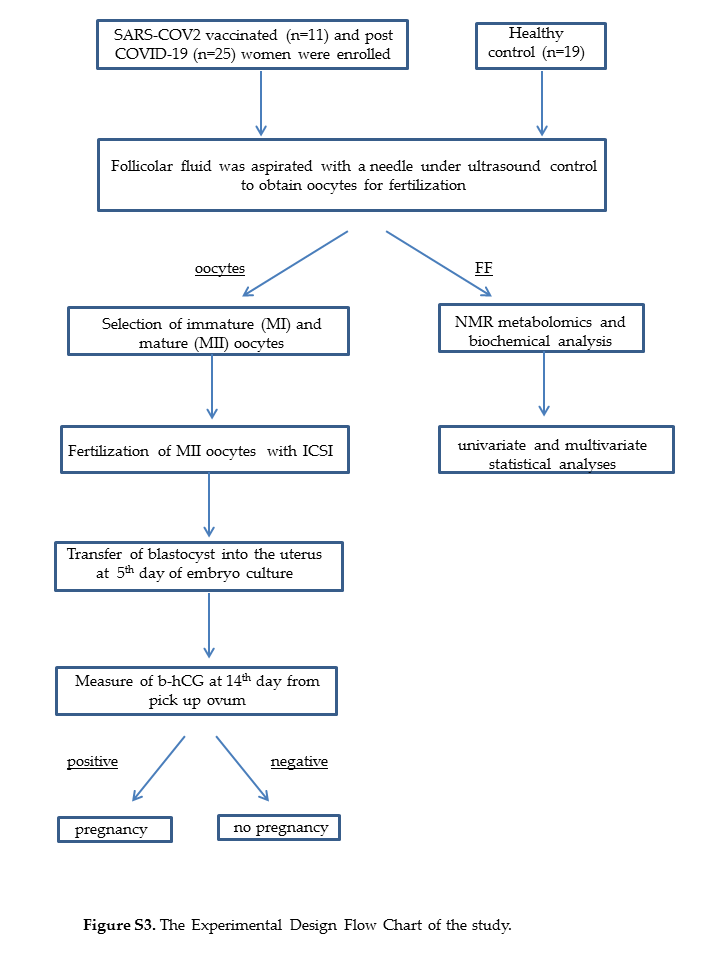

Supplement: Supplementary file 1 [file ijms-25-08400-s001.zip › Figure S3.tif]
